# The characteristic ultrasound features of specific types of ovarian pathology (Review)

**DOI:** 10.3892/ijo.2014.2764

**Published:** 2014-11-18

**Authors:** AHMAD SAYASNEH, CHRISTINE EKECHI, LAURA FERRARA, JEROEN KAIJSER, CATRIONA STALDER, SHYAMALY SUR, DIRK TIMMERMAN, TOM BOURNE

**Affiliations:** 1 Department of Cancer and Surgery, Imperial College London, Hammersmith Campus, London, W12 0HS, UK; 2 Early Pregnancy and Acute Gynecology Unit, Queen Charlottes and Chelsea Hospital, Imperial College London, London, W12 0HS, UK; 3 Department of Development and Regeneration, KU Leuven, Leuven, Belgium

**Keywords:** ovarian cancer, ovarian neoplasm, ovary, pattern recognition, ultrasonography

## Abstract

Characterizing ovarian masses enables patients with malignancy to be appropriately triaged for treatment by subspecialist gynecological oncologists, which has been shown to optimize care and improve survival. Furthermore, correctly classifying benign masses facilitates the selection of patients with ovarian pathology that may either not require intervention, or be suitable for minimal access surgery if intervention is required. However, predicting whether a mass is benign or malignant is not the only clinically relevant information that we need to know before deciding on appropriate treatment. Knowing the specific histology of a mass is becoming of increasing importance as management options become more tailored to the individual patient. For example predicting a mucinous borderline tumor gives the opportunity for fertility sparing surgery, and will highlight the need for further gastrointestinal assessment. For benign disease, predicting the presence of an endometrioma and possible deeply infiltrating endometriosis is important when considering both who should perform and the extent of surgery. An examiner’s subjective assessment of the morphological and vascular features of a mass using ultrasonography has been shown to be highly effective for predicting whether a mass is benign or malignant. Many masses also have features that enable a reliable diagnosis of the specific pathology of a particular mass to be made. In this narrative review we aim to describe the typical morphological features seen on ultrasound of different adnexal masses and illustrate these by showing representative ultrasound images.

## 1. Introduction

The characterization of ovarian masses and distinguishing between benign and malignant pathology is important both to decrease unnecessary anxiety and enable decisions regarding optimal treatment. Benign pathology may be best treated conservatively or in a general gynecology unit using a minimal access approach. Conversely, suspected malignant masses should be referred to specialized units for further management. Thus prior knowledge of the nature of ovarian masses is essential not only for the patient but in order to organize clinical services in terms of planning, costs and overall management (1).

Transvaginal ultrasonography (TVS) is the most commonly employed imaging modality for the assessment of adnexal masses, and a number of prediction models have been created to maximize its predictive capability. In many countries the risk of malignancy index (RMI) (2) which combines ultrasound features, serum CA125 levels and the menopausal status of the patient is still used to characterize ovarian pathology. However, more recently logistic regression models and simple rules created by the International Ovarian Tumor Analysis (IOTA) group have been shown to perform better than the RMI (3–7). The most recent systematic review and meta-analysis has concluded that based on currently available evidence, these IOTA rules and models should now be used in clinical practice (3). Notwithstanding these advances, the optimal approach to characterizing ovarian masses remains the subjective interpretation of the ultrasound features of a mass by an expert operator (8–10).

For the purposes of this review, the term ‘pattern recognition’ refers to the subjective evaluation of adnexal masses using grey-scale and power/color Doppler ultrasonography (11,12). In the hands of experienced examiners pattern recognition has a high sensitivity (77–86%) and specificity (94–100%) to diagnose teratomas/dermoid cysts, endometriomas, hydrosalpinges and peritoneal pseudocysts (13). It has however, not been found to be as useful for the diagnosis of fibromas, paraovarian cysts and rare benign tumors, and may have difficulty in differentiating between physiological and other ‘simple’ cysts on the basis of a single scan (sensitivity 8–17%) (13).

These findings suggest that with adequate training and knowledge of the common features associated with particular pathologies, ultrasound examiners should be able to reliably diagnose and differentiate between certain specific types of adnexal pathology. It is important to remember that when evaluating women with an adnexal mass, ultrasound characteristics need to be correlated with the clinical history, as well as signs and symptoms before arriving at a diagnosis. This review describes only the features that may be found using ultrasound that may be used to predict common specific types of adnexal pathology.

## 2. Physiological, peritoneal and tubal cystic pathology

### Follicular cysts

**Figure 1:**
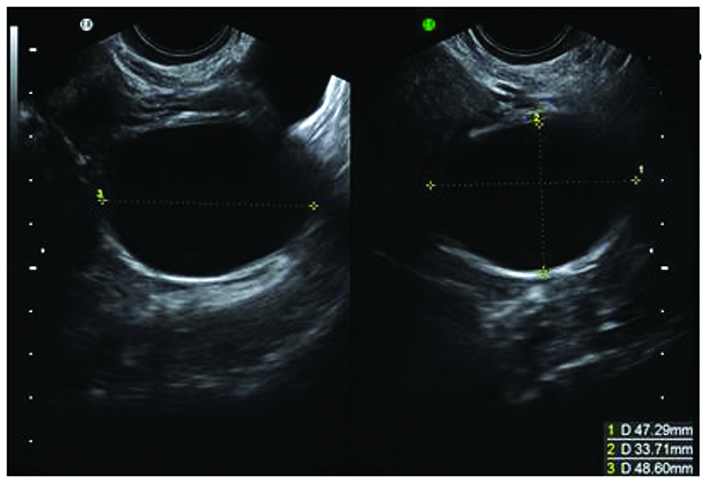
Follicular ‘physiological’ cyst. Note the bright white hyperechoic posterior wall enhancement.

They are usually unilocular and thin walled with anechoic contents (12). They rarely exceed 8–10 cm in diameter and typically spontaneously resolve within 6 weeks (14). Posterior wall hyperechoic enhancement is a feature due to reflection of the ultrasound beam off the posterior wall having travelled through the anechoic window formed by the clear cyst contents (14) (Fig. 1).

### Corpus luteum cysts

**Figure 2:**
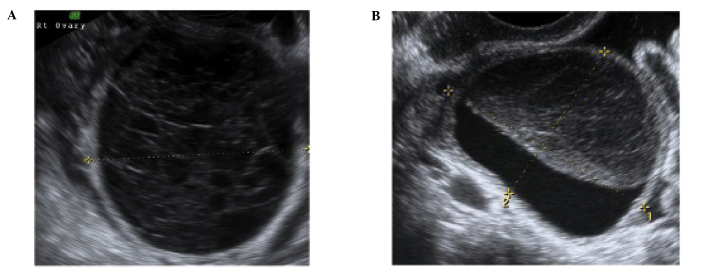
The cob-web sign, which represents the fibrin strings of a recently formed clot within a hemorrhagic corpus luteum cyst (A), and after clot retraction (B).

These are formed following the rupture of a mature Graafian follicle. They are thick walled hyperechoic cysts that typically demonstrate peripheral circumferential blood flow, sometimes known as the ‘ring of fire’ (12). Some cysts may show areas of internal hemorrhage. The cyst contents typically have a spider-web-like appearance (Fig. 2) due to a small amount of internal hemorrhage, but can frequently show different features including blood clots within the cyst resembling solid components. Doppler examination may be useful in these circumstances as the blood clot will have no blood flow, although perhaps more useful is the a typical jelly-like ‘wobbling’ movement that can be elicited from the blood clot within the cyst if the vaginal probe is used to gently prod the ovary during the examination (15). In most cases, hemorrhagic cysts resolve within 6–12 weeks without intervention (15).

### Peritoneal pseudocysts

Peritoneal pseudocysts, are collections of peritoneal fluid trapped in adhesions usually caused by previous pelvic surgery, pelvic inflammatory disease or endometriosis. They usually occur in premenopausal women, because of the presence of functional ovaries that release small amounts of fluid into the peritoneal cavity (15–18). They grow gradually and may reach several centimeters in size. They can cause abdominal pain or distension, but in the majority of cases are asymptomatic (15–18).

**Figure 3:**
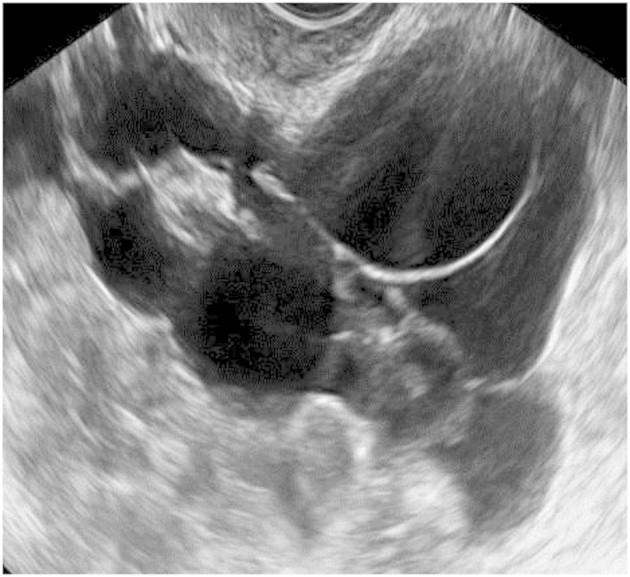
Multilocular peritoneal inclusion cysts.

Pseudocysts appear mainly as multilocular cysts, with a high number of septa that are adherent to the ovarian surface. Septa are most frequently complete and thin (15–18) (Fig. 3). In contrast to septae within true ovarian cysts the septae in pseudocysts generally move and ‘flap’ when the cystic area is prodded by the transvaginal ultrasound probe. This has been described as the ‘flapping sail sign’ (18). They have an irregular shape, that follows the contours of the pouch of Douglas or pelvic sidewall and surrounding pelvic organs, giving a ‘lumpy’, ‘star-like’ or ‘tubular’ appearance (15–18).

**Figure 4:**
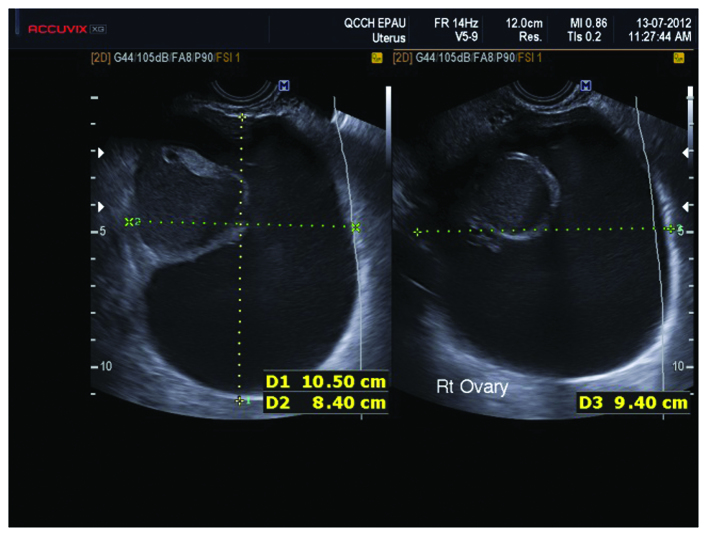
A non-septated peritoneal pseudocyst with the ovary seen separately containing an endometrioma and follicles in the cortex. The patient has a clinical history of multiple surgical procedures for endometriosis.

The ipsilateral ovary is visible in almost all cases (Fig. 4). It can be external to the lesion or entrapped within the cyst (17,18). The cyst contents are generally anechoic, but may show low-level echogenicity (16,18).

### Paraovarian cysts

**Figure 5:**
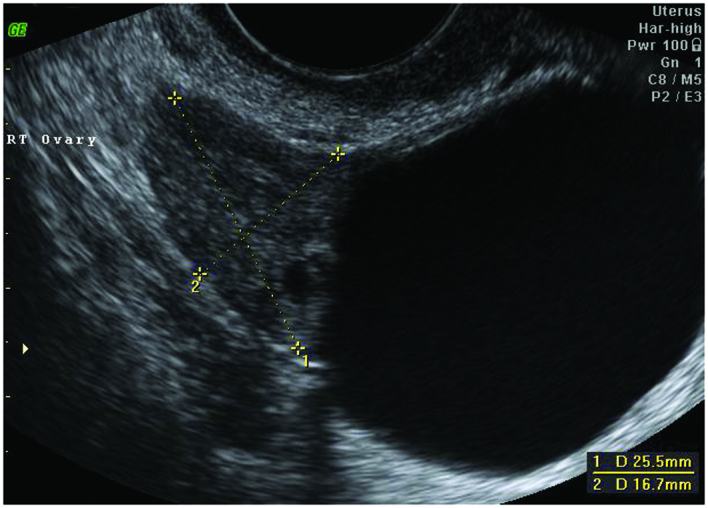
A paraovarian cyst with a normal ovary seen separate to it.

Paraovarian cysts arise in the broad ligament between the ovary and the fallopian tube. They account for 5–20% of adnexal masses (19,20). The incidence of borderline and malignant paraovarian tumors is low but cases have been reported (20,21). They appear as thin walled unilocular anechoic masses close to but separate from the ovary (Fig. 5). However they can show papillary projections in ~30% of cases (20).

Their mean diameter is usually <5 cm with no evidence of any follicles or significant vascularity. In almost all cases, it is possible to visualize the ipsilateral normal ovary, and to detect movement of the cyst in the opposite direction to the ovary when the area is pushed with the vaginal probe - the ‘split sign’. This may help to differentiate between a paraovarian and ovarian cyst when the ipsilateral ovary is not clearly visible (20).

### Tubal pathology

**Figure 6:**
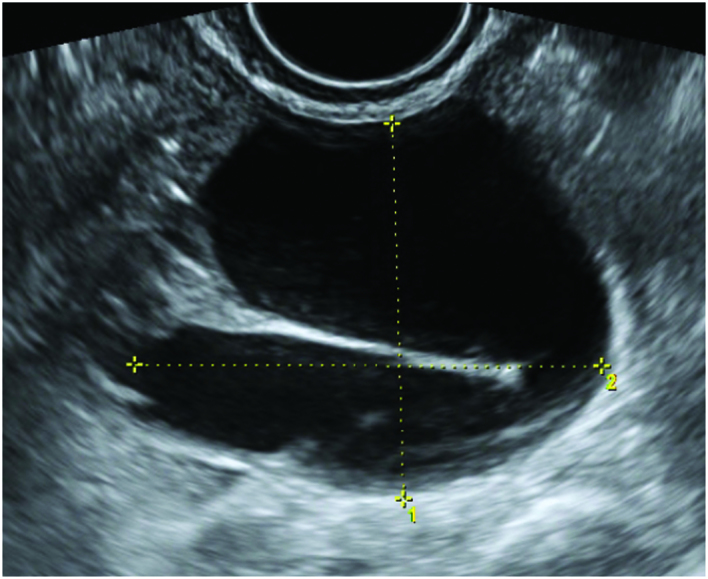
Incomplete septum in a hydrosalpinx.

A normal Fallopian tube is rarely visible during an ultrasound examination. Hydrosalpinges have typical diagnostic features on ultrasound with anechoic contents and incomplete septae (15) (Fig. 6). In the case of an acute or chronic inflammatory process the tube may become detectable and some specific characteristics have been described.

**Figure 7:**
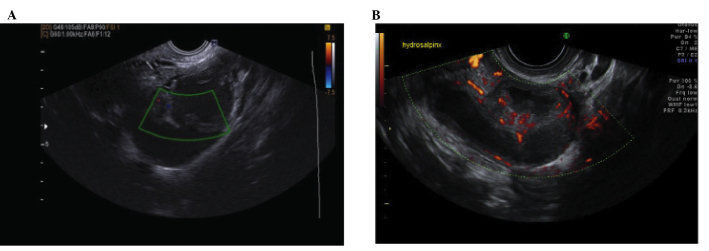
Acute salpingitis demonstrating incomplete septae and thick walls. (A) An example of increased vascularity in an incomplete septum using color Doppler TVS. (B) Another example using power Doppler TVS.

**Figure 8:**
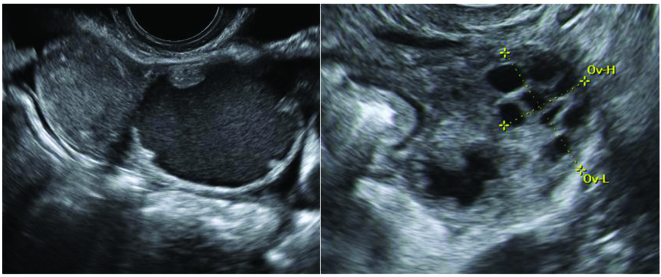
The cogwheel sign.

Acute salpingitis typically appears like a pear-shaped unilocular mass with anechoic or low-level content, characterized by thickening of the wall (>5 mm) and the presence of incomplete septae (Fig. 7). In transverse section it often shows the well described ‘cogwheel sign’ appearance (15,22) (Fig. 8). Color or power Doppler examination generally shows significant vascularity in cases of an acute inflammatory process as well as the presence of fluid in the pouch of Douglas (23).

In chronic salpingitis the tube appears as an elongated fluid-filled mass, with incomplete septae, but the thickening of the wall is no longer visible. It is characterized by the typical sonographic ‘beads on a string’ sign, due to 2–3 mm sized hyperechoic structures on the tubal wall, seen on transverse section (15,22–24).

**Figure 9:**
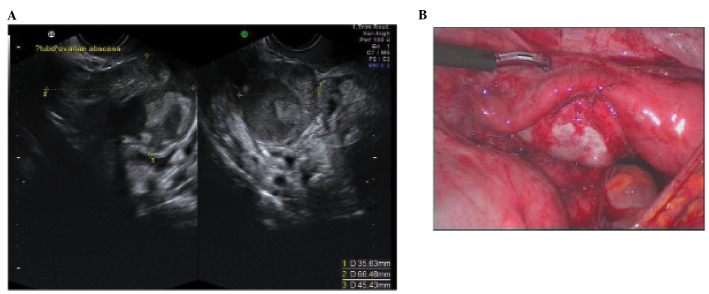
A tubo-ovarian complex. (A) Ultrasound appearances. (B) The same case at laparoscopy.

A tubo-ovarian complex represents the involvement of ovarian tissue in the inflammatory process. Normal ovarian parenchyma is visible, but it is usually seen separate from tubal structures (15,22–24) (Fig. 9).

In a tubo-ovarian abscess, ovarian tissue is no longer visible; the lesion may be unilocular, solid or multilocular-solid with mixed or ground-glass echogenicity. On the basis of the ultrasound features, these have to be differentiated from endometriomas or hemorrhagic cysts (15,22–24). In practice the clinical features associated with an abscess make the diagnosis relatively straightforward.

## 3. Ovarian pathology

### Serous cystadenomas

**Figure 10:**
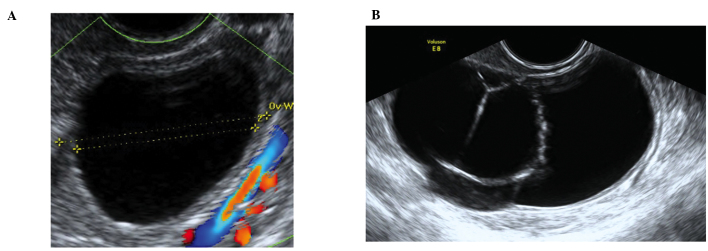
Serous cystadenoma. (A) Unilocular serous cystadenoma. (B) Multilocular cystadenoma.

These appear as smooth, thin walled, anechoic, fluid-filled structures. They are bilateral in 15% of cases and their mean size is 5–8 cm (25). Some contain fine septations whilst others have areas of haemorrhage appearing as small echogenic areas (25) (Fig. 10).

### Mucinous cystadenomas

**Figure 11:**
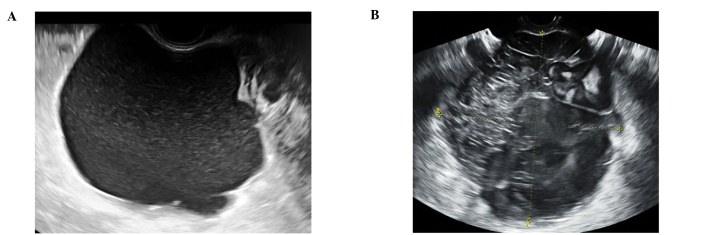
Mucinous cystadenomas. (A) Unilocular. (B) Multilocular.

Mucinous cysts are classically thin walled, large and unilateral. They consist of internal thin-walled locules containing mucin which appears as fluid with low level echogenicity (25) (Fig. 11). In general neither serous nor mucinous cystadenomas are associated with significant vascularity (25).

**Figure 12:**
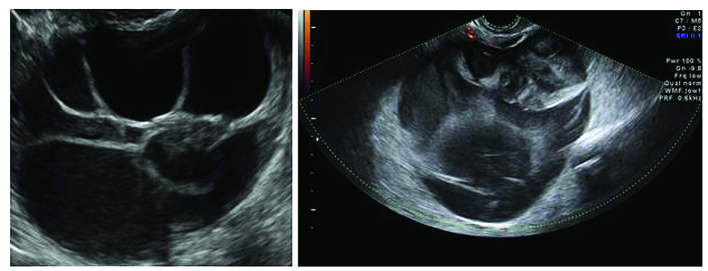
A mucinous cystadenoma with variable echogenicity among the cyst locules.

Caspi *et al* described the presence of variable echogenicity among different tumor locules as an ultrasound feature of multilocular mucinous cystadenomas (26) (Fig. 12), however this has not been confirmed in larger studies to date.

### Cystadenofibromas

**Figure 13:**
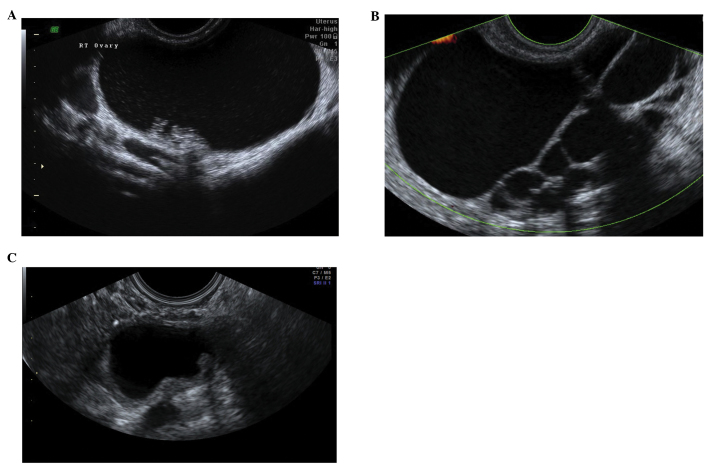
Serous cystadenofibromas. (A) Unilocular solid with a papillary projection and acoustic shadows. (B) Multilocular solid. (C) Another example of serous cystadenofibroma with unilocular solid morphology.

Cystadenofibromas represent a relatively rare type of benign epithelial ovarian tumor. They are mainly serous although mucinous subtypes do exist (27). Descriptions of the sonographic features of cystadenofibromas are limited but some specific appearances have been described. They may appear as unilocular-solid, or less frequently, multilocular-solid masses with thin cyst walls and anechoic contents (15,27). The diagnosis may be aided by the presence of hyperechoic solid components with acoustic shadows and low to moderate vascularity (15,27). They are often seen as unilocular-solid lesions with single papillary projections. The key feature to look for then is acoustic shadowing even within these small papillations (15,27). Differentiating between cystadenofibromas and borderline or malignant ovarian masses can be difficult (15,27) (Fig. 13).

### Mature teratoma/dermoid cysts

**Figure 14:**
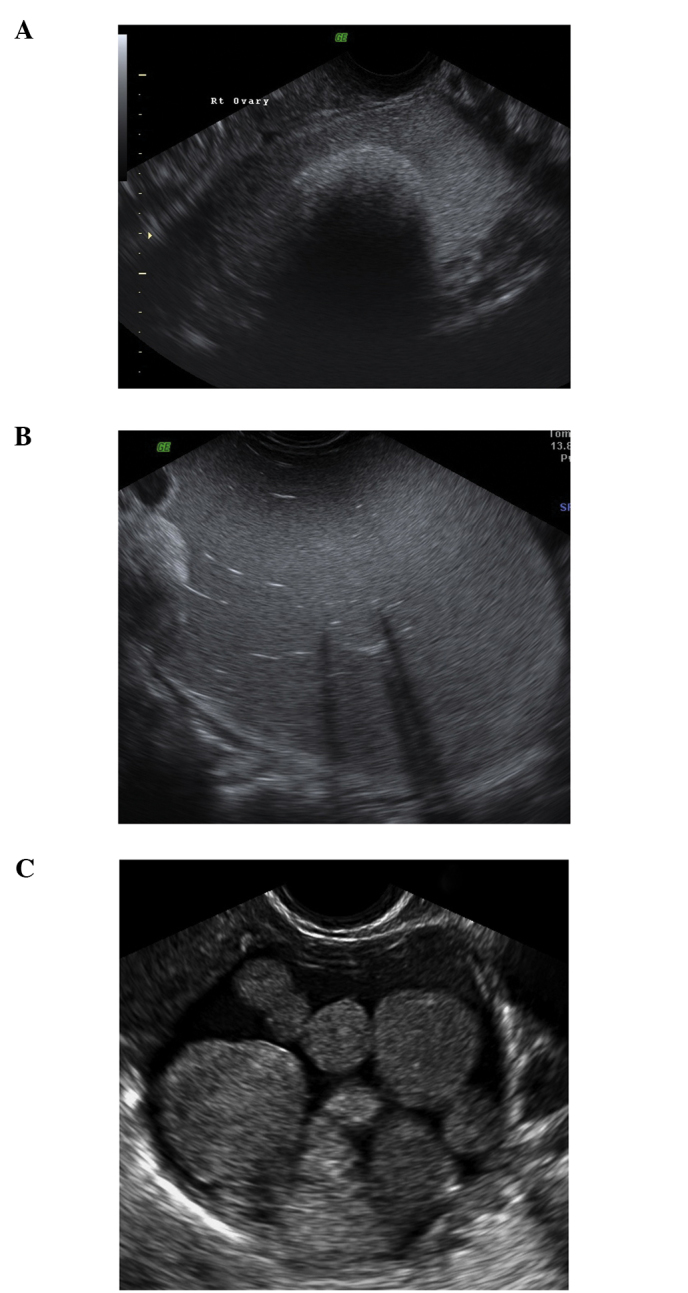
Ultrasound features of dermoid cysts. (A) Rokitansky nodule with a strong acoustic shadow. (B) Acoustic shadows and bright echoes representing hair in the cyst. (C) Unusual but interesting presentation of a dermoid cyst which has been described as ‘floating balls’ - secondary to hyperechoic intracystic fat balls.

Mature cystic teratomas are benign germ cell tumors. They usually have the highest sensitivity and specificity for a specific diagnosis with ultrasound as they generally have rather typical features (28). They are cystic and unilocular in the majority of cases, with mixed echogenicity representing the different components of fat, bone and fluid (28). Pathognomonic of dermoid cysts is a Rokitansky nodule, a distinct hyperechoic mural nodule representing areas of floating hair in low-density fluid (29,30) (Fig. 14). There are often bright echoes and sharp acoustic shadows associated with hair or even teeth in the cyst.

### Endometriomas

**Figure 15:**
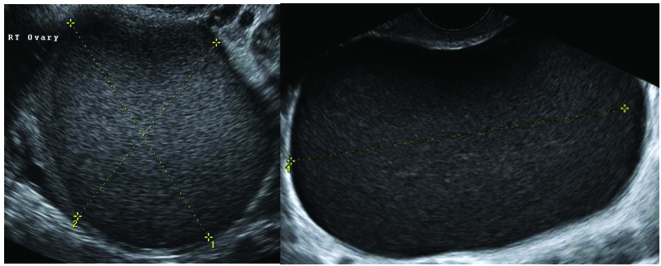
Typical endometriomas.

Ultrasonography is particularly sensitive for accurately diagnosing ‘typical’ endometriomas, most commonly seen in premenopausal women. Typically an endometrioma is a unilocular tumor and has low-level echogenicity representing old blood in the cyst cavity (commonly termed ‘ground glass’). It is this ‘ground glass’ feature that is the most typical feature (28,31–33) (Fig. 15).

**Figure 16:**
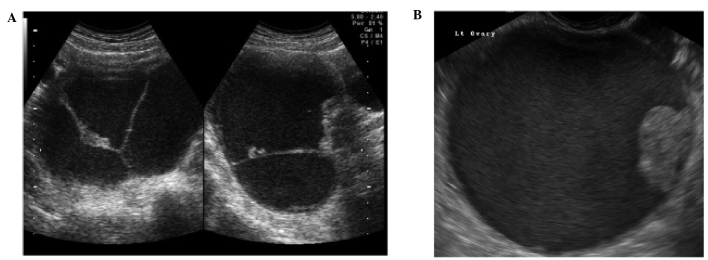
Atypical endometriomas with solid papillary projections. (A) Multilocular solid endometrioma. (B) Unilocular solid endometrioma.

Endometriomas may also have atypical features, and frequently debris within the cyst may give the impression that it is a unilocular-solid lesion with solid papillary projections. In postmenopausal women the appearances of an atypical endometrioma should be examined very carefully as there is a significant risk of malignancy in such lesions in this age group (29,32) (Fig. 16).

**Figure 17:**
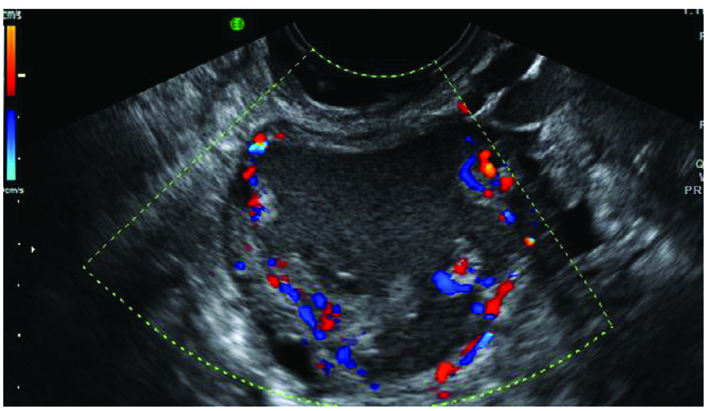
Decidualized endometrioma in pregnancy with vascularized papillary projections.

During pregnancy endometriomas can change their appearance secondary to decidualization. The features may become quite alarming, with solid vascular projections into the cyst cavity. When no pre-existing scan of the ovary is documented it is difficult in these cases not to suspect malignancy (Fig. 17), although papillary projections were a more frequent sonographic feature among malignant lesions than among benign endometrioid cysts (34,35).

### Ovarian fibromas and fibrothecomas

These are benign tumors of stromal origin. Fibromas originate from spindle cells producing collagen and can be associated with ascites or Meig’s syndrome. Fibrothecomas originate from both spindle and theca cells and may produce a small amount of estrogens (36,37).

**Figure 18:**
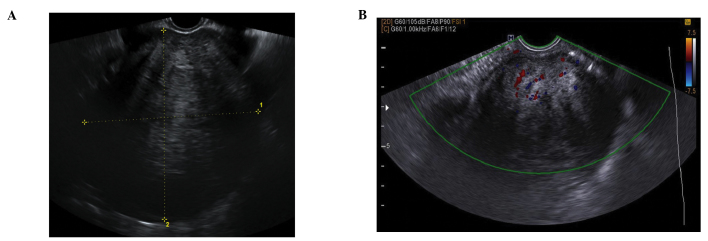
Typical round regular ovarian fibroma with (A) acoustic shadows and (B) minimal peripheral vascularity on color Doppler.

**Figure 19:**
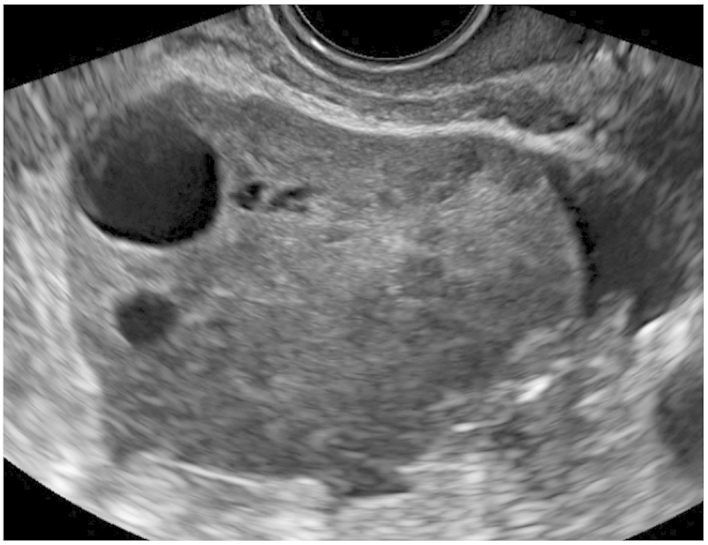
Ovarian fibroma with cystic changes.

Their characteristic sonographic appearance is of a round or oval solid tumor, with regular margins. They may have stripy acoustic shadows, but these are present in just a small percentage of cases (15,36,37) (Fig. 18). Fibromas and fibrothecomas can also show cystic areas, due to hemorrhage, edema or necrosis within the stromal tissue (Fig. 19). Doppler findings are variable, but frequently the lesions show little peripheral vascularity (36,37) (Fig. 18).

### Ovarian stromal tumors (struma ovarii)

**Figure 20:**
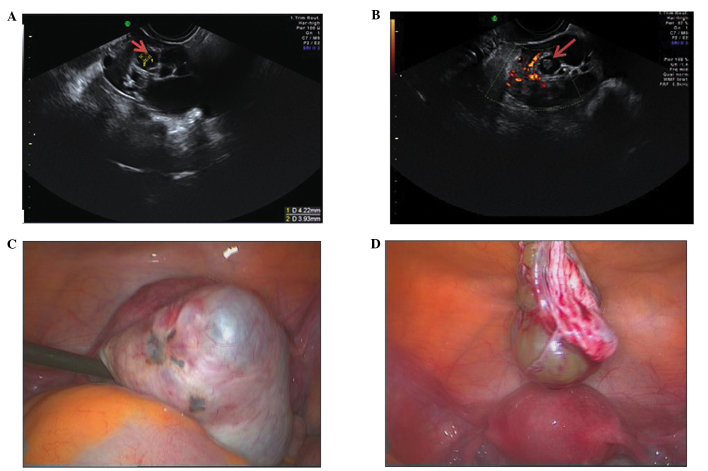
Struma ovarii showing (A) multilocularity and struma pearl formation (arrow) as well as (B) central vascularity (arrow pointing toward the ‘pearl’). (C and D) Laparoscopic features of the same cyst at the time of cystectomy.

Struma ovarii is a rare subtype of mature teratoma characterized by the presence of ectopic thyroid tissue. They account for <5% of mature teratomas (38). Although a preoperative diagnosis is not always possible, they have been described as having a similar appearances to mature teratomas but with increased vascularity in the central part of the mass (39). They are difficult to classify (40), but are of interest morphologically because they have been associated with a sonographic sign called the ‘struma pearl’. These are rounded hyperechogenic structures with smooth surfaces, with increased vascularity on Doppler examination (40) (Fig. 20).

### Brenner tumors

**Figure 21:**
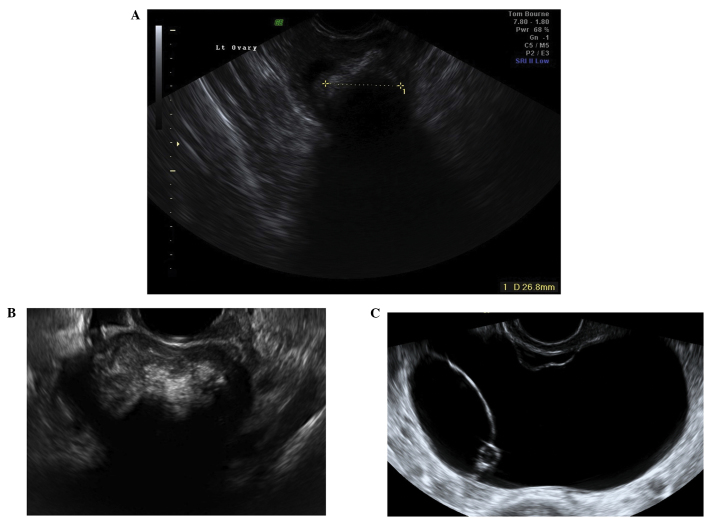
Brenner tumors. (A and B) Solid Brenner tumor with marked acoustic shadowing. (C) Brenner tumor with mucinous cystadenoma.

Brenner tumors also arise from the ovarian stroma but are benign in 99% of cases. Their diagnosis is often an incidental finding in women between the fifth and the seventh decade of life. They are usually small and often coexist with serous or mucinous cystadenomas (Fig. 21). They are more frequently unilateral, mainly within the left ovary (41–43). Brenner tumors are sometimes associated with acoustic shadowing and so may be confused with an ovarian fibroma or pedunculated fibroid from the uterus (Fig. 21) (41–43).

### Primary invasive ovarian epithelial cancer

**Figure 22:**
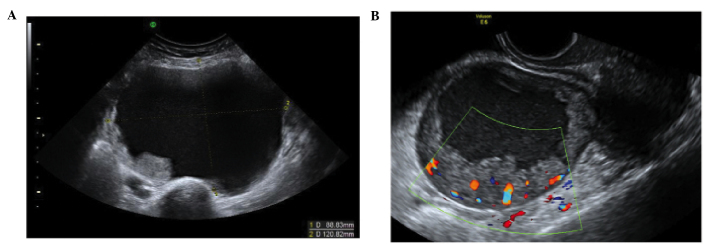
Primary invasive ovarian epithelial cancers. (A) Stage 1 clear cell carcinoma of the ovary. (B) Unilocular solid early invasive cancer with increased vascularity on color Doppler.

Stage 1 primary invasive ovarian epithelial cancers share similar ultrasound characteristics to borderline tumors, but they differ significantly from the appearances of later stage disease (44) (Fig. 22). They often contain papillary projections and less commonly are purely solid (44).

**Figure 23:**
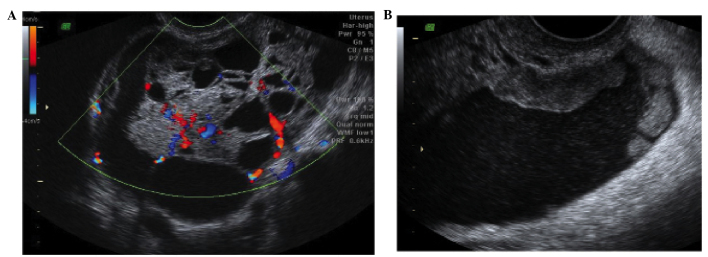
Advanced primary ovarian cancers. (A) Multilocular solid ovarian serous adenocarcinoma with increased vascularity. (B) Peritoneal deposits from late stage primary ovarian cancer in in the pouch of Douglas with ascites.

Later stage primary ovarian tumors are usually multilocular with a high proportion of solid tissue and are frequently associated with ascites as well as metastatic disease to the peritoneum, omentum and elsewhere in the abdomen and pelvis (44). They are also significantly vascular with high color scores (3–4) (44) (Fig. 23).

### Borderline tumors

**Figure 24:**
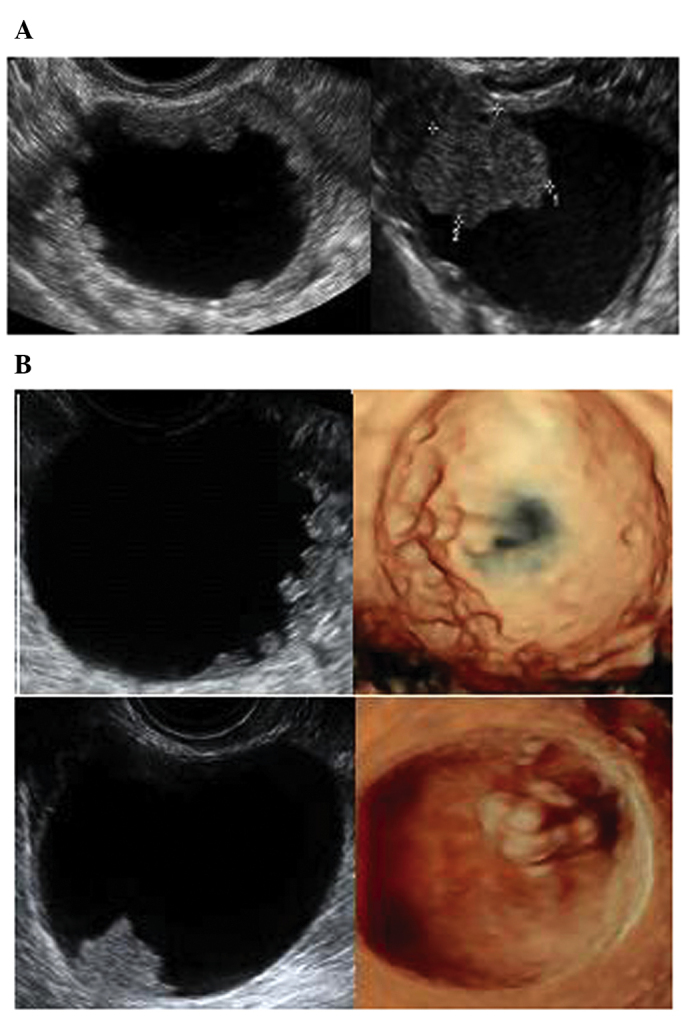
Ovarian serous borderline tumors. (A) Papillary projection with irregular surface. (B) Papillary projections in cases of serous BOT with their 3D images.

**Figure 25:**
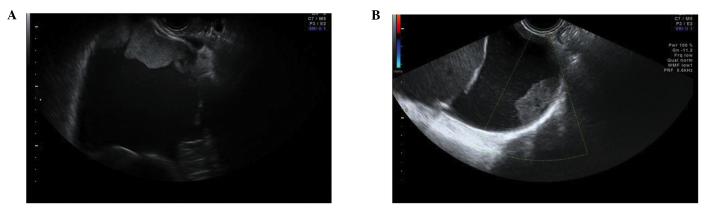
Mucinous endocervical BOT. (A) B mode image. (B) Color Doppler image.

**Figure 26:**
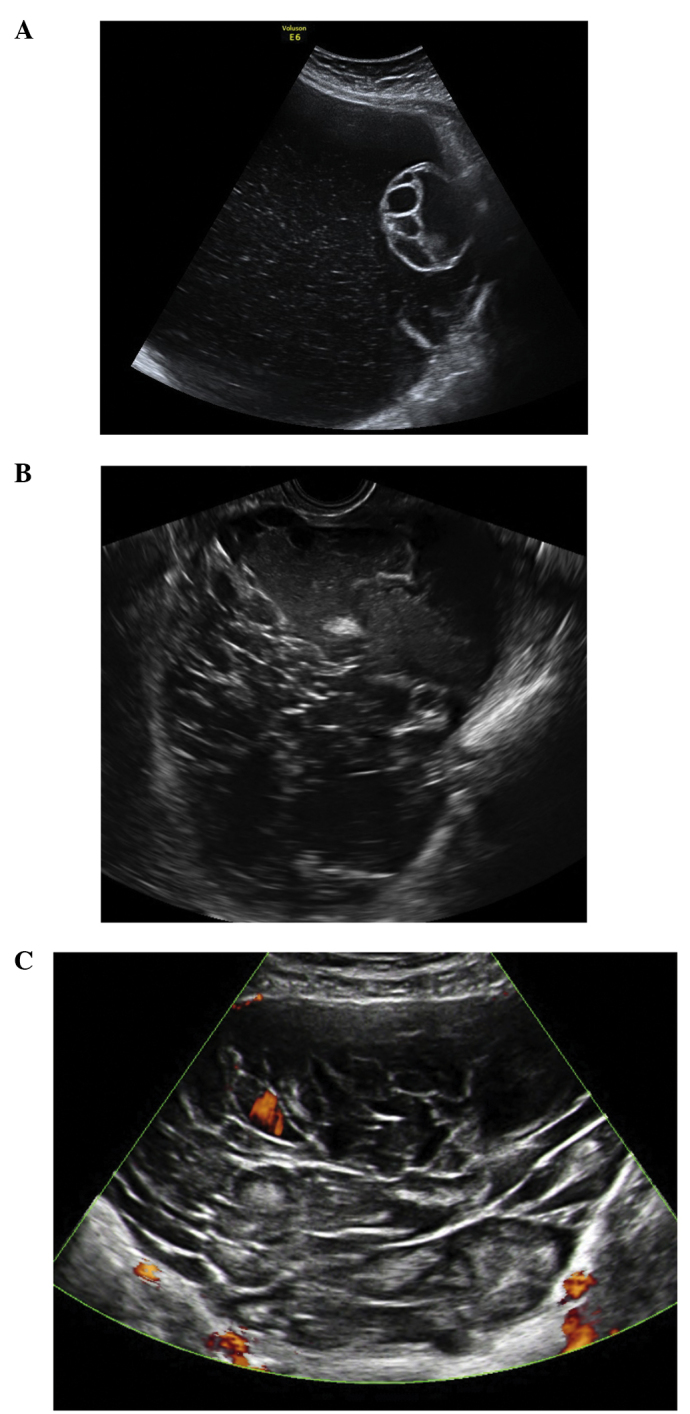
Mucinous intestinal BOTs. (A) Honeycomb or cribriform sign. (B and C) Intense multilocularity in intestinal type mucinous BOT.

The presence of papillary projections within a cyst has been used as a discriminatory factor for serous borderline tumors (45). However, the potential for misdiagnosis between borderline tumors (BOT), cystadenomas, cystadenofibromas and invasive malignant tumors is significant (45). Doppler assessment of tumor vascularity is not useful in distinguishing between borderline and invasive tumors (45,46). The size and characteristics of the surface of the papillary projections are however thought to be helpful with the angle the projection makes with the cyst wall being significantly different (47) (Figs. 24–26). In this review the mean size of papillary projections was 9.6, 15.7, and 35.3 mm in benign, borderline, and malignant tumors, respectively. In benign masses an acute angle was present between the cyst wall and projection in 68% of cases and an obtuse angle in 40% of borderline and 89% when the mass was an invasive malignancy. These observations are of interest, but have not yet been validated in larger prospective studies (47).

Serous and mucinous endocervical type BOTs are usually unilocular solid tumors with a high number of vascular papillary projections within the cyst. Mucinous intestinal type BOT are more often very large, unilateral, multilocular tumors with a high number of locules encased by thick, hyperechoic tissue with no evidence of solid components (Figs. 24–26). They are associated with the ‘honeycomb’ sign formed by tightly interrelated septae within the cyst. Intestinal-type mucinous BOT are generally less vascular than both serous and endocervical BOT (48,49).

### Tumors that have metastasized to the ovary

**Figure 27:**
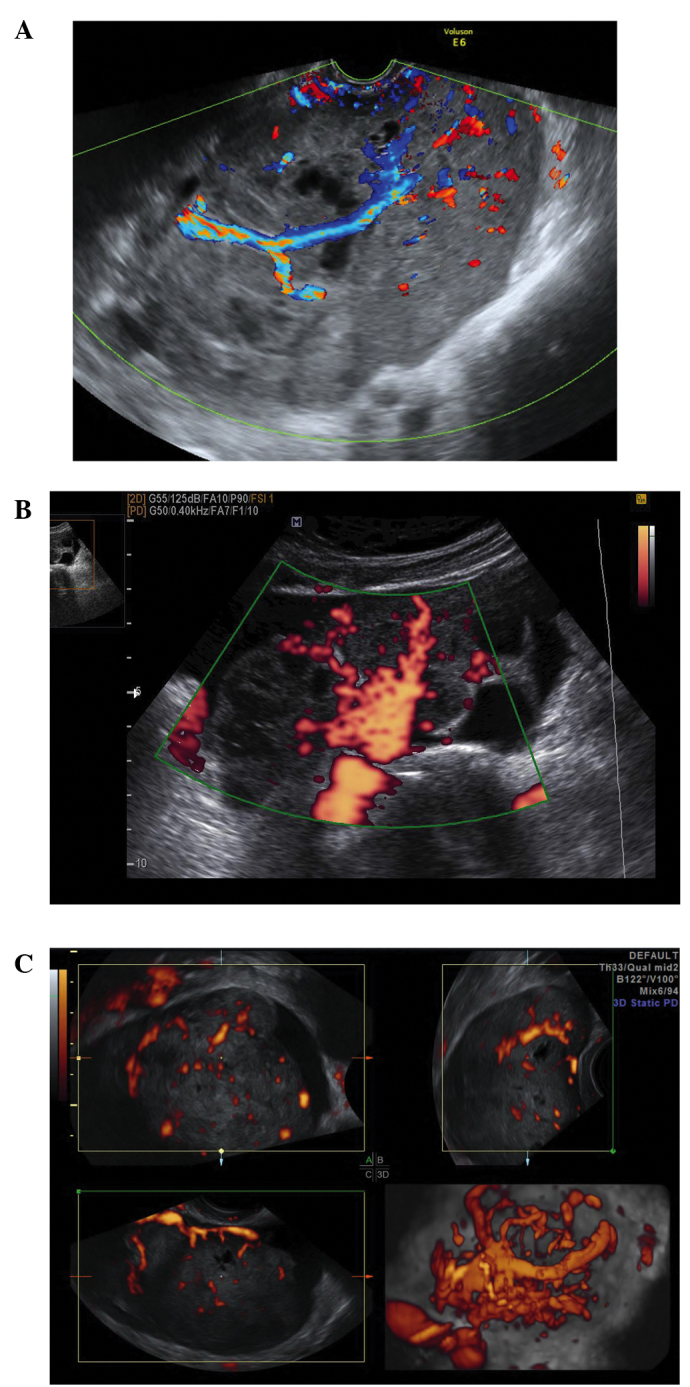
Breast cancer with metastasis to the ovaries. (A) Lead vessel sign in color Doppler 2D image. (B) Lead vessel sign in power Doppler 2D image. (C) Lead vessel sign in 3D power Doppler image.

**Figure 28:**
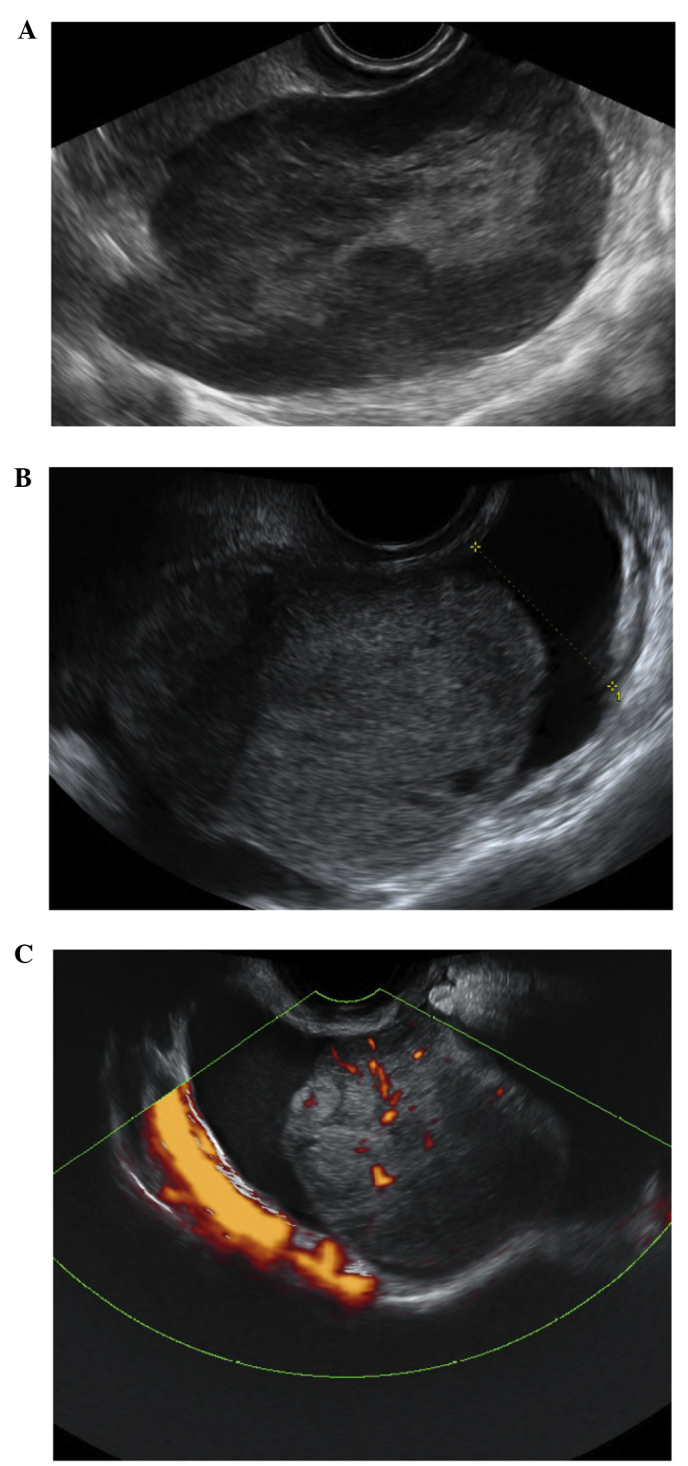
Metastatic cancers to the ovary appear as solid tumors. (A) Lymphoma. (B) Gastric adenocarcinoma. (C) Gastric adenocarcinoma with metastasis to the ovary with using power Doppler 2D image.

**Figure 29:**
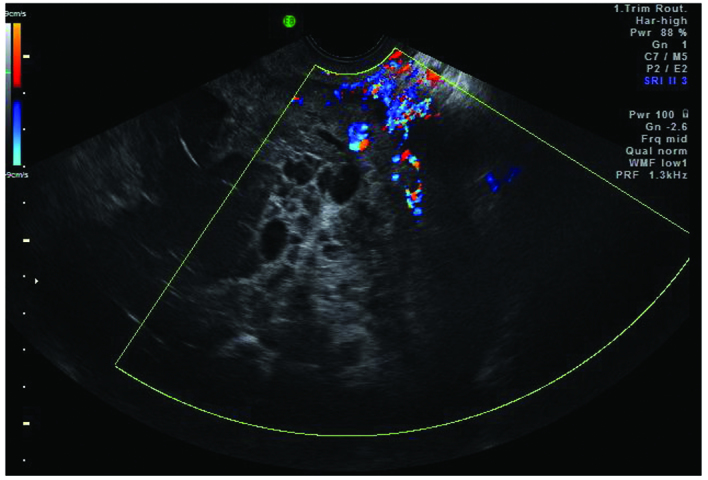
Colon cancer with metastasis to the ovary.

**Figure 30:**
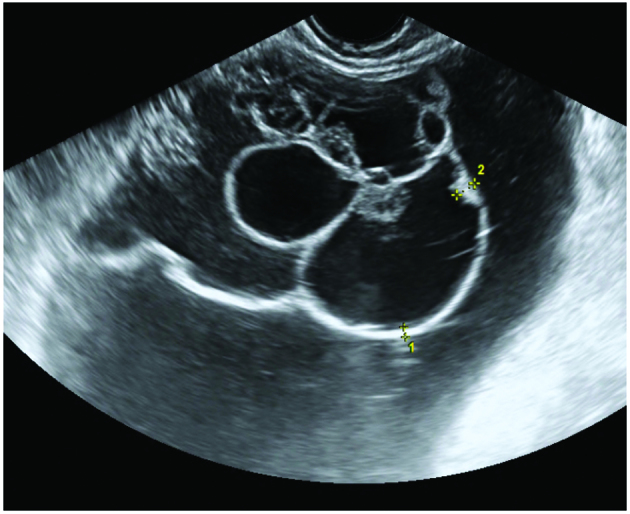
Pancreatic cancer with metastasis to the ovaries.

Ovarian metastasis from breast, gastric, and uterine cancers as well as lymphomas appear as solid tumors on ultrasound examination (Figs. 27 and 28). In contrast, ovarian metastasis from the colon, rectum and biliary tract, tend to be multilocular-solid or multilocular with anechoic or low-level echogenicity (50) (Figs. 29 and 30). The latter group demonstrate, a larger diameter and more frequently the presence of an irregular external surface (50). The detection of papillary projections is rare in metastatic tumors (50) (Figs. 27–30). The presence of rich vascularity (color score 3–4) is characteristic of all metastatic tumors (44), but metastatic tumors from the colon, rectum and biliary tract tend to be less vascular compared to those from the stomach, breast, uterus or lymphomas (50).

The vascularity of metastatic tumors is characterized by the presence of a ‘lead vessel’ - a single large vessel penetrating from the periphery to the central part of the lesion (Fig. 27). Further research is needed to determine the diagnostic performance of this sign (51).

### Conclusion

Predicting the specific histopathology of an adnexal mass is important as it may lead to surgery being avoided or being less invasive in some cases whilst ensuring appropriate referral to a gynecological oncology surgeon in the case of malignancy. In general there is an intense focus on excluding malignancy when the characterization of ovarian pathology is considered. However the field has moved on, both in terms of tailoring treatment to individual patients and with what we know about the features of different types of ovarian pathology. In this review we hope we have illustrated some of the pathognomonic features of some of the more commonly found adnexal masses in clinical practice. By improving the specific classification of masses we hope that management decisions in relation to such pathology will become more patient specific and lead to improved outcomes.

## References

[1] Carley ME, Klingele CJ, Gebhart JB, Webb MJ, Wilson TO (2002). Laparoscopy versus laparotomy in the management of benign unilateral adnexal masses. J Am Assoc Gynecol Laparosc.

[2] Jacobs I, Oram D, Fairbanks J, Turner J, Frost C, Grudzinskas JG (1990). A risk of malignancy index incorporating CA 125, ultrasound and menopausal status for the accurate preoperative diagnosis of ovarian cancer. Br J Obstet Gynaecol.

[3] Kaijser J, Sayasneh A, Van Hoorde K (2014). Presurgical diagnosis of adnexal tumours using mathematical models and scoring systems: a systematic review and meta-analysis. Hum Reprod Update.

[4] Sayasneh A, Wynants L, Preisler J (2013). Multicentre external validation of IOTA prediction models and RMI by operators with varied training. Br J Cancer.

[5] Timmerman D, Van Calster B, Testa AC (2010). Ovarian cancer prediction in adnexal masses using ultrasound-based logistic regression models: a temporal and external validation study by the IOTA group. Ultrasound Obstet Gynecol.

[6] Timmerman D, Ameye L, Fischerova D (2010). Simple ultrasound rules to distinguish between benign and malignant adnexal masses before surgery: prospective validation by IOTA group. BMJ.

[7] Testa A, Kaijser J, Wynants L (2014). Strategies to diagnosie ovarian cancer: new evidence from phase 3 of the multicentre international IOTA study. Br J Cancer.

[8] Valentin L, Ameye L, Savelli L (2011). Adnexal masses difficult to classify as benign or malignant using subjective assessment of gray-scale and Doppler ultrasound findings: logistic regression models do not help. Ultrasound Obstet Gynecol.

[9] Timmerman D, Schwarzler P, Collins WP (1999). Subjective assessment of adnexal masses with the use of ultrasonography: an analysis of interobserver variability and experience. Ultrasound Obstet Gynecol.

[10] Timmerman D (2004). The use of mathematical models to evaluate pelvic masses; can they beat an expert operator?. Best Pract Res Clin Obstet Gynaecol.

[11] Valentin L, Hagen B, Tingulstad S, Eik-Nes S (2001). Comparison of ‘pattern recognition’ and logistic regression models for discrimination between benign and malignant pelvic masses: a prospective cross validation. Ultrasound Obstet Gynecol.

[12] Valentin L (1999). Pattern recognition of pelvic masses by gray-scale ultrasound imaging: the contribution of Doppler ultrasound. Ultrasound Obstet Gynecol.

[13] Sokalska A, Timmerman D, Testa AC (2009). Diagnostic accuracy of transvaginal ultrasound examination for assigning a specific diagnosis to adnexal masses. Ultrasound Obstet Gynecol.

[14] Jeong YY, Outwater EK, Kang HK (2000). Imaging evaluation of ovarian masses. Radiographics.

[15] Valentin L (2004). Use of morphology to characterize and manage common adnexal masses. Best Pract Res Clin Obstet Gynaecol.

[16] Kurachi H, Murakami T, Nakamura H (1993). Imaging of peritoneal pseudocysts: value of MR imaging compared with sonography and CT. AJR Am J Roentgenol.

[17] Jain KA (2000). Imaging of peritoneal inclusion cysts. AJR Am J Roentgenol.

[18] Savelli L, de Iaco P, Ghi T, Bovicelli L, Rosati F, Cacciatore B (2004). Transvaginal sonographic appearance of peritoneal pseudocysts. Ultrasound Obstet Gynecol.

[19] Dorum A, Blom GP, Ekerhovd E, Granberg S (2005). Prevalence and histologic diagnosis of adnexal cysts in postmenopausal women: an autopsy study. Am J Obstet Gynecol.

[20] Savelli L, Ghi T, De Iaco P, Ceccaroni M, Venturoli S, Cacciatore B (2006). Paraovarian/paratubal cysts: comparison of transvaginal sonographic and pathological findings to establish diagnostic criteria. Ultrasound Obstetrics Gynecol.

[21] Smorgick N, Herman A, Schneider D, Halperin R, Pansky M (2009). Paraovarian cysts of neoplastic origin are underreported. JSLS.

[22] Timor-Tritsch IE, Lerner JP, Monteagudo A, Murphy KE, Heller DS (1998). Transvaginal sonographic markers of tubal inflammatory disease. Ultrasound Obstet Gynecol.

[23] Romosan G, Bjartling C, Skoog L, Valentin L (2013). Ultrasound for diagnosing acute salpingitis: a prospective observational diagnostic study. Hum Reprod.

[24] Guerriero S, Ajossa S, Lai MP, Mais V, Paoletti AM, Melis GB (2000). Transvaginal ultrasonography associated with colour Doppler energy in the diagnosis of hydrosalpinx. Hum Reprod.

[25] Karlan BY, Bristow RE, Li AJ (2012). Gynecologic Oncology: Clinical Practice & Surgical Atlas.

[26] Caspi B, Hagay Z, Appelman Z (2006). Variable echogenicity as a sonographic sign in the preoperative diagnosis of ovarian mucinous tumors. J Ultrasound Med.

[27] Alcazar JL, Errasti T, Minguez JA, Galan MJ, Garcia-Manero M, Ceamanos C (2001). Sonographic features of ovarian cystadenofibromas: spectrum of findings. J Ultrasound Med.

[28] Ameye L, Timmerman D, Valentin L (2012). Clinically oriented three-step strategy for assessment of adnexal pathology. Ultrasound Obstet Gynecol.

[29] Jermy K, Luise C, Bourne T (2001). The characterization of common ovarian cysts in premenopausal women. Ultrasound Obstet Gynecol.

[30] Cohen L, Sabbagha R (1993). Echo patterns of benign cystic teratomas by transvaginal ultrasound. Ultrasound Obstet Gynecol.

[31] Guerriero S, Ajossa S, Mais V, Risalvato A, Lai MP, Melis GB (1998). The diagnosis of endometriomas using colour Doppler energy imaging. Hum Reprod.

[32] Van Holsbeke C, Van Calster B, Guerriero S (2010). Endometriomas: their ultrasound characteristics. Ultrasound Obstet Gynecol.

[33] Asch E, Levine D (2007). Variations in appearance of endometriomas. J Ultrasound Med.

[34] Sayasneh A, Naji O, Abdallah Y, Stalder C, Bourne T (2012). Changes seen in the ultrasound features of a presumed decidualised ovarian endometrioma mimicking malignancy. J Obstet Gynaecol.

[35] Testa AC, Timmerman D, Van Holsbeke C (2011). Ovarian cancer arising in endometrioid cysts: ultrasound findings. Ultrasound Obstet Gynecol.

[36] Yen P, Khong K, Lamba R, Corwin MT, Gerscovich EO (2013). Ovarian fibromas and fibrothecomas: sonographic correlation with computed tomography and magnetic resonance imaging: a 5-year single-institution experience. J Ultrasound Med.

[37] Paladini D, Testa A, Van Holsbeke C, Mancari R, Timmerman D, Valentin L (2009). Imaging in gynecological disease (5): clinical and ultrasound characteristics in fibroma and fibrothecoma of the ovary. Ultrasound Obstet Gynecol.

[38] Roth LM, Talerman A (2007). The enigma of struma ovarii. Pathology.

[39] Zalel Y, Seidman DS, Oren M (2000). Sonographic and clinical characteristics of struma ovarii. J Ultrasound Med.

[40] Savelli L, Testa AC, Timmerman D, Paladini D, Ljungberg O, Valentin L (2008). Imaging of gynecological disease (4): clinical and ultrasound characteristics of struma ovarii. Ultrasound Obstet Gynecol.

[41] Green GE, Mortele KJ, Glickman JN, Benson CB (2006). Brenner tumors of the ovary: sonographic and computed tomographic imaging features. J Ultrasound Med.

[42] Sherer DM, Dalloul M, Salame G (2009). Color Doppler sonographic features of a Brenner tumor in pregnancy. J Ultrasound Med.

[43] Dierickx I, Valentin L, Van Holsbeke C (2012). Imaging in gynecological disease (7): clinical and ultrasound features of Brenner tumors of the ovary. Ultrasound Obstet Gynecol.

[44] Valentin L, Ameye L, Testa A (2006). Ultrasound characteristics of different types of adnexal malignancies. Gynecol Oncol.

[45] Exacoustos C, Romanini ME, Rinaldo D (2005). Preoperative sonographic features of borderline ovarian tumors. Ultrasound Obstet Gynecol.

[46] Pascual MA, Tresserra F, Grases PJ, Labastida R, Dexeus S (2002). Borderline cystic tumors of the ovary: gray-scale and color Doppler sonographic findings. J Clin Ultrasound.

[47] Hassen K, Ghossain MA, Rousset P (2011). Characterization of papillary projections in benign versus borderline and malignant ovarian masses on conventional and color Doppler ultrasound. AJR Am J Roentgenol.

[48] Fruscella E, Testa AC, Ferrandina G (2005). Ultrasound features of different histopathological subtypes of borderline ovarian tumors. Ultrasound Obstet Gynecol.

[49] Darai E, Teboul J, Walker F (1996). Epithelial ovarian carcinoma of low malignant potential. Eur J Obstet Gynecol Reprod Biol.

[50] Testa AC, Ferrandina G, Timmerman D (2007). Imaging in gynecological disease (1): ultrasound features of metastases in the ovaries differ depending on the origin of the primary tumor. Ultrasound Obstet Gynecol.

[51] Testa AC, Mancari R, Di Legge A (2008). The ‘lead vessel’: a vascular ultrasound feature of metastasis in the ovaries. Ultrasound Obstet Gynecol.

